# Nitrous oxide consistently attenuates thermogenic and thermoperceptual responses to repetitive cold stress in humans

**DOI:** 10.1152/japplphysiol.00309.2023

**Published:** 2023-07-20

**Authors:** Maaike I. Moes, Antonis Elia, Mikael Gennser, Ola Eiken, Michail E. Keramidas

**Affiliations:** Division of Environmental Physiology, Swedish Aerospace Physiology Center, KTH Royal Institute of Technology, Stockholm, Sweden

**Keywords:** hypothermia, inert gas narcosis, shivering thermogenesis, thermoeffector plasticity, thermoregulation

## Abstract

Divers are at enhanced risk of hypothermia, due to the independent action of the inspired inert gases on thermoregulation. Thus, narcosis induced by acute (≤2 h) exposure to either hyperbaric nitrogen or normobaric nitrous oxide (N_2_O) impairs shivering thermogenesis and accelerates body core cooling. Animal-based studies, however, have indicated that repeated and sustained N_2_O administration may prevent N_2_O-evoked hypometabolism. We, therefore, examined the effects of prolonged intermittent exposure to 30% N_2_O on human thermoeffector plasticity in response to moderate cold. Fourteen men participated in two ∼12-h sessions, during which they performed sequentially three 120-min cold-water immersions (CWIs) in 20°C water, separated by 120-min rewarming. During CWIs, subjects were breathing either normal air or a normoxic gas mixture containing 30% N_2_O. Rectal and skin temperatures, metabolic heat production (via indirect calorimetry), finger and forearm cutaneous vascular conductance (CVC; laser-Doppler fluxmetry/mean arterial pressure), and thermal sensation and comfort were monitored. N_2_O aggravated the drop in rectal temperature (*P* = 0.01), especially during the first (by ∼0.3°C) and third (by ∼0.4°C) CWIs. N_2_O invariably blunted the cold-induced elevation of metabolic heat production by ∼22%–25% (*P* < 0.001). During the initial ∼30 min of the first and second CWIs, N_2_O attenuated the cold-induced drop in finger (*P* ≤ 0.001), but not in forearm CVC. N_2_O alleviated the sensation of coldness and thermal discomfort throughout (*P* < 0.001). Thus, the present results demonstrate that, regardless of the cumulative duration of gas exposure, a subanesthetic dose of N_2_O depresses human thermoregulatory functions and precipitates the development of hypothermia.

**NEW & NOTEWORTHY** Human thermoeffector plasticity was evaluated in response to prolonged iterative exposure to 30% N_2_O and moderate cold stress. Regardless of the duration of gas exposure, N_2_O-induced narcosis impaired in a persistent manner shivering thermogenesis and thermoperception.

## Graphical abstract

**Figure:**
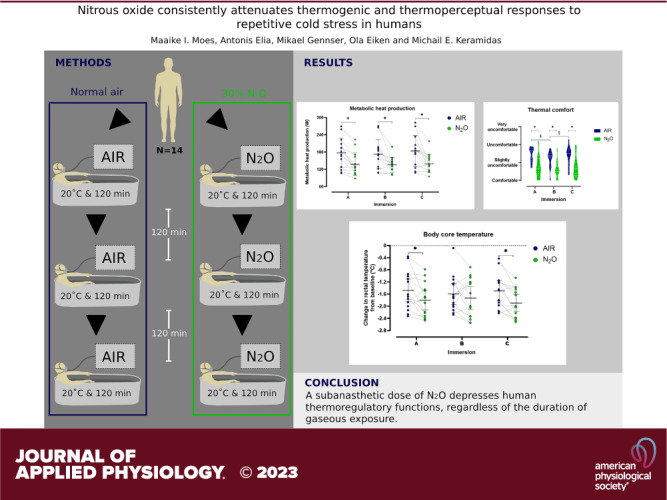

## INTRODUCTION

At high ambient pressures, human thermoregulatory capacity to cold is perturbed by the inert gases inspired, predisposing divers to enhanced risk of accidental hypothermia. Specifically, mild narcosis engendered by hyperbaric air attenuates shivering thermogenesis and the perceived magnitude of thermal discomfort, and accelerates body core cooling (1). These thermoadaptive modifications have been attributable to the independent action of the increased partial pressure of nitrogen (N_2_) on the function of one or several thermoregulatory control element(s), namely, the thermosensory transduction, the conveyance and integration of the sensory inputs, and/or the activity of the thermoeffector end-organs (for review, see Ref. 2).

In a manner alike to that of hyperbaric N_2_, inhalation of nitrous oxide (N_2_O) at atmospheric pressure also exerts an influence on thermoregulation. Specifically, short-term (≤2 h) exposure to ≤30% N_2_O blunts thermogenic and thermoperceptual responsiveness to cold, and precipitates hypothermia (3–5). Similar N_2_O-evoked alterations have been confirmed in other species, in which, along with the metabolic and thermo-behavioral attenuations, the heat-conservation processes are also downregulated (6, 7). It is noteworthy, however, that, in homeothermic animals (e.g., rats), long-term sustained or repeated exposure to N_2_O may prevent the hypothermic response prompted by acute N_2_O, and may, by contrast, eventually augment heat-producing and heat-dissipating thermoeffectors; though thermal behavior is persistently impaired (8–12). The mechanism(s) underlying such time-dependent sensitization of the autonomic thermoeffectors is unknown, but it may be orchestrated through gradual modulations of the central neural circuitry occurring over successive N_2_O provocations.

The present study, therefore, aimed to examine human thermoregulatory plasticity in response to iterative exposures to N_2_O and cold. For this purpose, autonomic and perceptual responses were evaluated during fixed moderate-intensity cold stimulation (i.e., 2-h immersion in 20°C water) used three times serially within a day (13, 14), while subjects were breathing either normal air or a normoxic gas mixture containing 30% N_2_O. On the basis of the aforementioned animal studies (8–12), we hypothesized that prolonged exposure to N_2_O would gradually diminish the metabolic, but not the thermoperceptual impairments incurred by acute N_2_O during cold.

## METHODS

### Ethics Approval

The study was approved by the Regional Human Ethics Committee in Stockholm (Ref. No.: 2021-05314-01) and conformed to the standards set by the Declaration of Helsinki (except for registration in a database). Before participation, written informed consent was obtained from all subjects.

### Subjects

Based on a previous work using a similar experimental design to the present (14), a minimum sample size of 12 individuals was determined a priori, using α = 0.05, β = 0.85, and an effect size of *f* = 0.31 (G*power software, Heinrich Heine-Universitat, Düsseldorf, Germany; 15). In total, 16 healthy men were recruited. Two subjects, however, withdrew from the study, due to adverse effects (e.g., severe nausea and discomfort) encountered during the first 30 min of breathing N_2_O—in both cases, the trial was terminated immediately after reporting the symptoms, and subjects were fully recovered within ∼5 min after commencing to breathe room air. We, therefore, included 14 subjects, with the following characteristics: mean (range) age 23 (18–28) yr, height 179 (164–192) cm, weight 71.4 (55.7–93.6) kg, body surface area 1.90 (1.10–2.18) m^2^, body fat 8.3 (4.1–12.6)%, and total skinfold thickness 64 (35–91) mm. Considering that autonomic thermoregulation (16) and thermal perception (17) may be influenced by female reproductive hormones, we did not recruit any woman for this study. Subjects were free of any cardiorespiratory, hematological, or metabolic disease, had no history of cold injuries, and were vaccinated against SARS-CoV-2 (Covid-19). An exclusion criterion was a history or family history of vitamin B_12_ deficiency because N_2_O inhibits the enzyme methionine synthetase and inactivates vitamin B_12_, which may lead to neurologic deterioration (18, 19). Subjects were not smokers or teetotallers, nor habitual users of N_2_O (5 of them, however, had inhaled N_2_O for recreational purposes, at least once before their recruitment to the study). All resided in the Stockholm region for ≥6 mo before the initiation of the study, were physically active on a recreational basis, and were not exposed regularly to cold water nor did they perform diving-related activities. Subjects were also instructed to avoid any severe cold exposure during the study period.

### Study Design

**Figure 1.**
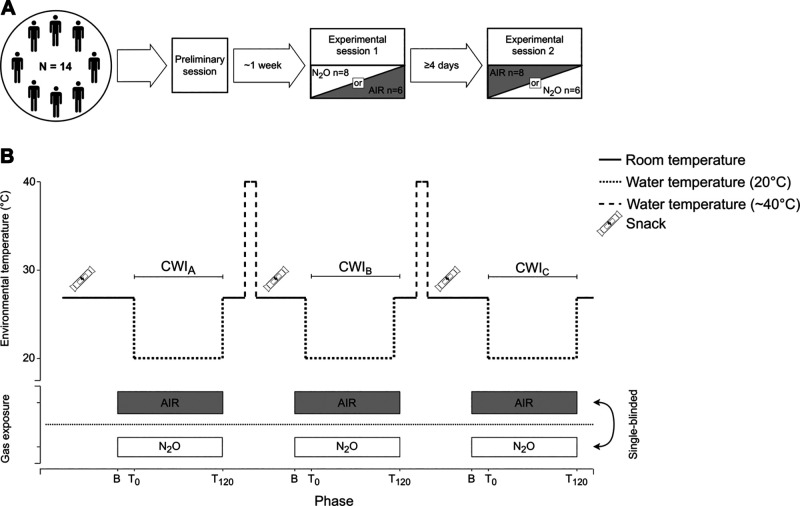
Overview of the overall study design (*A*) and of the experimental protocol of the two 12-h main sessions (*B*). AIR, normal air; B, 20-min baseline phase; CWI_A_, 1st cold-water immersion; CWI_B_, 2nd cold-water immersion; CWI_C_, 3rd cold-water immersion; N_2_O, normoxic gas mixture containing 30% nitrous oxide; T_0_, start of the cold-water immersion; T_120_, end of the 120-min cold-water immersion (note: the immersion was terminated prematurely, if the rectal temperature dropped ≤35°C).

The study utilized a repeated-measures, single-blinded design. Thus, all subjects attended a preliminary session and two main ∼12-h experimental sessions (Fig. 1*A*). During the main sessions, subjects performed three 120-min cold (20°C) water immersions (CWIs; 1st CWI: CWI_A_, 2nd CWI: CWI_B_, and 3rd CWI: CWI_C_), separated by a 120-min rewarming period. During each CWI, subjects were breathing, through an oronasal mask, either normal air (AIR) or a normoxic gas mixture containing 30% N_2_O. The use of the specific concentration of N_2_O was based on previous evidence showing that, in healthy humans, acute (≤120 min) exposure to such levels impairs thermogenic and thermoperceptual responses to cold (4, 5). All sessions were performed between February and July. Yet, for the individual subject, the two main sessions were conducted within a 5-wk period and were separated by at least 4 days, to eliminate the potential induction of cold habituation. The order of the sessions was alternated among subjects: eight and six of them performed first the N_2_O and AIR session, respectively. Subjects were asked to abstain from alcohol and heavy exercise during the 24-h period preceding the main experimental sessions. Also, to ensure that similar nutritional and exercise routines were maintained before the main sessions, subjects were asked to keep diet and exercise logs for 48 h before the sessions.

#### Preliminary session.

Approximately a week before the first main session, subjects attended the preliminary session, during which they underwent a medical examination and were familiarized with the experimental procedure. Anthropometric measures were also performed. Body weight and height were measured with an electronic scale (Vetek, Väddö, Sweden) and a stadiometer, respectively. The body surface area was derived from the weight and height measures (20). Skinfold thicknesses were assessed with a caliper (Harpenden, West Sussex, UK) at seven sites on the right side of the body: triceps, subscapular, chest, suprailiac, abdominal, front thigh, and midaxillary. The equation of Jackson and Pollock (21) was subsequently used to calculate the body fat percentage.

#### Main experimental sessions.

The experimental protocol is depicted in Fig. 1*B*. Subjects arrived at the laboratory at ∼0700 h, after an overnight fast. They were accustomed to the ambient conditions of the laboratory [mean (standard deviation; SD) temperature and barometric pressure were 27.3 (0.3)°C and 1,014 (11) mmHg, respectively] for ∼60 min, while the instrumentation was conducted. During this period, subjects ate a standardized snack (see below for details) and drank 250 mL water—in case their urine specific gravity, assessed with a refractometer (PAL-105, ATAGO, Tokyo, Japan), was >1.025, they were asked to consume 250 mL water additionally. After emptying their bowel and bladder, subjects’ body weight was measured.

Thereafter, subjects, who were always dressed in swim-shorts, assumed a resting semireclined position on a gurney adjacent to the immersion tank. They were equipped with an oronasal mask and inspired either AIR or N_2_O. Each CWI commenced with a 20-min baseline phase. Then, subjects were immersed, within a ≤5-s period, to the level of the xiphoid process in a tank containing 20°C water. They remained in a semiupright sitting position, with both arms being supported at the level of the heart above the water surface, for up to 120 min, or until the rectal temperature (T_rec_) fell below 35°C. Subjects were instructed to avoid any voluntary movements and were free to watch movies throughout. The water in the tank was stirred constantly; its temperature was monitored continuously by two thermistors (PT100, Texas Instrument, Dallas, Texas), and, if necessary, adjusted by adding cold or warm water.

CWI_A_ and CWI_B_ were followed by a 120-min rewarming phase, during which T_rec_ returned to the CWI_A_ baseline values. The oronasal mask was removed after the end of each CWI (i.e., subjects were breathing room air throughout the rewarming phases) and was put back just before the initiation of the baseline phase. The rewarming phase consisted of a 30-min passive rewarming on the gurney, while subjects were placed in a plastic-lined sleeping bag, followed by a ∼30-min active rewarming (i.e., whole body immersion in ∼40°C water), and again by passive rewarming until the next baseline phase. After CWI_C_, subjects took a warm shower, were toweled, and their body weight was measured.

Upon arrival at the laboratory, and after each active rewarming phase, subjects were asked to consume a 55-g protein bar (6.8 g fat, 17 g carbohydrates, 20 g protein; Barebells Functional Foods AB, Stockholm, Sweden). While eating the snack, subjects were also provided with 250 mL water; yet they were free to drink more if desired. The total energy [AIR session: 553 (52) kcal, N_2_O session: 557 (51) kcal; *P* = 0.32] and water [AIR session: 858 (196) mL, N_2_O session: 897 (242) mL; *P* = 0.48] intakes were similar in the two sessions. During the rewarming phase, subjects urinated in a urinal bottle, when needed. The total amount of the collected urinary output did not differ between sessions [AIR session: 1,507 (377) mL, N_2_O session: 1,446 (496) mL; *P* = 0.77].

### Instrumentation

#### Thermometry.

T_rec_ was measured using a thermistor probe (Yellow Springs Instruments, Yellow Springs, OH) inserted 10 cm beyond the anal sphincter. Mean skin temperature (T_sk_) was derived from the unweighted average of skin temperatures, which were monitored with copper-constantan (T-type) thermocouple probes (Physitemp Instruments Inc, Clifton, NJ) placed at the foot, calf, thigh, abdomen, upper arm, forearm, finger, and forehead. All temperatures were sampled at 1 Hz with a NI USB-6215 data acquisition system and processed with LabVIEW software (version 2019, National Instruments, Austin, TX).

#### Respiratory measurements.

During the baseline and CWIs, subjects breathed through a low-resistance two-way respiratory valve (model 2, 700 T-Shape, Hans Rudolph, Shawnee, KS). The inspiratory side of the valve was connected via respiratory corrugated tubing to a bag filled with the premixed humidified breathing gas. Inspiratory minute ventilation (V̇i) was monitored with a turbine ventilation module (KL Engineering, Los Angeles, CA). Expired air was directed through respiratory hosing to a 10-l Plexiglas mixing box. A sample of the expired air was drawn continuously from the mixing box at a rate of 0.2 L·min^−1^ and was analyzed for oxygen (Applied Electrochemistry model S-3A/I, Pittsburgh, PA) and carbon dioxide (Beckman model LB-2, Fullerton, CA) contents. Before each session, the pneumotachograph and the gas analyzers were calibrated with a 3-l syringe and two different gas mixtures (gas-1: 20.93% O_2_ and 0.04% CO_2_; gas-2: 16.0% O_2_ and 5.0% CO_2_), respectively. Because N_2_O may alter the absorption of infrared by CO_2_ due to the “collision broadening effect” (22), and may also influence the detection of O_2_, the analyzers in the N_2_O session were calibrated with gas mixtures containing 30% N_2_O in combination with O_2_, CO_2_, and N_2_.

All respiratory values were sampled at 1 Hz with a data acquisition system (LabVIEW, National Instruments). Off-line analysis was performed using a custom-made computer program based on LabVIEW (National Instruments). Oxygen uptake (V̇o_2_; expressed in L·min^−1^), carbon dioxide production (V̇co_2_; expressed in L·min^−1^), respiratory exchange ratio (RER), and V̇i (expressed in L·min^−1^) were calculated at 60-s intervals. The individual shivering thresholds, indicated by a sustained elevation (>0.10 L·min^−1^) in V̇o_2_, were determined from the responses of V̇o_2_ relative to changes in T_rec_ (ΔT_rec_; 4). Metabolic heat production (*Ṁ*; expressed in W) was estimated using: *Ṁ* = (0.23 × RER + 0.77) × 5.873V̇o_2_ × 60 (23). Energy expenditure (EE; expressed in kcal) was calculated using: EE = (3.94V̇o_2_ + 1.106V̇co_2_) × immersion duration (24).

#### Skin blood flux.

Laser-Doppler flowmetry (VMS-LDF2; Moor Instruments, Axminster, UK) was used to monitor local skin blood flux, at a rate of 1 Hz. The optic probes (VP1/7; Moor Instruments) were placed on the palmar side of the distal phalanx of the index finger and on the dorsal side of the forearm of the nondominant arm. Before each session, the probes were calibrated against Brownian motion with a standardized colloidal suspension of polystyrene microspheres. Skin blood flux was reported as absolute values of cutaneous vascular conductance [CVC; calculated as red blood cell flux divided by mean arterial pressure (MAP), and expressed in PU·mmHg^−1^].

#### Arterial pressures and heart rate.

Beat-to-beat systolic (SAP) and diastolic (DAP) arterial pressures and MAP were measured continuously using finger photoplethysmography (Finometer, Finapres Medical Systems BV, Enschede, the Netherlands). The pressure cuff was placed around the middle phalanx of the third finger of the nondominant hand, and the reference pressure transducer was positioned at the level of the heart. Before each CWI, a brachial cuff was attached on the same arm, and the calibration process was performed according to the manufacturer’s instructions. Heart rate (HR) was derived from the arterial pressure curves as the inverse of the interbeat interval.

#### Capillary glucose.

During the baseline and at *minute 100* (or, in case of early termination, just before exiting the tank) of each CWI, a finger capillary blood sample was drawn to assess glucose concentrations (Accu-Check, Aviva, Roche, Manheim, Germany).

#### Perceptual measurements.

During the baseline and at *minutes 1*, *30*, *60*, *90*, and *120* (or, in case of early termination, just before exiting the tank) of each CWI, subjects were asked to rate the whole body thermal sensation (from 1-cold to 7-hot) and comfort (from 1-comfortable to 4-very uncomfortable), the general affective valence (from −5-very bad to +5-very good), and the perceived shivering intensity (from 1-no shivering to 4-heavy shivering).

### Statistical Analyses

Baseline values were calculated as the average of the final 10 min of the 20-min baseline phase. Physiological data obtained during CWIs were averaged over 60 s. Because of unreliable forearm laser-Doppler signal in one subject, forearm CVC was reported for 13 subjects. Normality of distribution for all data was assessed using the Shapiro–Wilk test. Thermal, respiratory, and cardiovascular data were analyzed with two-way [breathing condition (AIR × N_2_O) × CWI (CWI_A_ × CWI_B_ × CWI_C_)] or three-way (breathing condition × CWI × time) repeated measures analysis of variance (ANOVA). Sphericity was assessed using Mauchly’s test, and the Greenhouse–Geiser ɛ correction was applied when necessary. When ANOVA revealed a significant *F* value, the Bonferroni correction was used to adjust for multiple post hoc comparisons. Kaplan–Meier survival curves and Mantel–Cox tests were used to assess differences in the duration of CWIs. Differences in the perceptual responses were determined with Friedman’s test, followed by the Wilcoxon signed-rank test. Student’s paired two-tailed *t* test was used to assess differences in body weight, urine output, and the amount of calorie and water intakes. Partial eta-squared (η_p_^2^) estimated the effect sizes, in which values of ≤0.02, ≤0.13, and ≥0.26 were considered small, moderate, and large, respectively. Cohen’s *d* assessed the effect sizes for the parametric pairwise-comparisons, in which values of ≤0.2, ≤0.5, and ≥0.8 were considered small, moderate, and large, respectively. The data visualization and statistical analyses were performed using SPSS (IBM Corp. Released 2020. IBM SPSS Statistics for Windows, version 27.0. Armonk, NY: IBM Corp) and Prism 9.0 (Graphpad Software Inc., San Diego, CA). Unless otherwise stated, data are presented as mean values with (SD). The α level of significance was set a priori at 0.05.

## RESULTS

In general, N_2_O engendered, at least initially, feelings of moderate euphoria. Subjects remained conscious throughout the N_2_O exposure but half of them experienced somnolence. Also, three subjects reported mild nausea, two reported lightheadedness, and one mentioned slight numbness in the fingers and toes. All, but one subject, identified the gas exposure correctly, when, at the end of each session, they were asked to guess whether they were breathing AIR or N_2_O.

On several occasions, the 120-min CWIs were terminated prematurely, because T_rec_ reached the end-point temperature of 35°C (4 subjects in the AIR session and 6 subjects in the N_2_O session). The immersion duration, however, did not differ between CWIs [mean duration (range): AIR session: CWI_A_: 114 (82–120) min, CWI_B_: 111 (74–120) min, CWI_C_: 110 (68–120) min; N_2_O session: CWI_A_: 107 (71–120) min, CWI_B_: 106 (74–120) min, CWI_C_: 105 (61–120) min; χ*^2^* = 1.78, *P* = 0.88].

In both the AIR and N_2_O sessions, baseline blood glucose was slightly lower (*P* ≤ 0.001) in CWI_A_ [AIR session: 5.4 (0.4) mmol·L^−1^, N_2_O session: 5.3 (0.4) mmol·L^−1^] than in CWI_B_ [AIR session: 5.8 (0.5) mmol·L^−1^, N_2_O session: 6.0 (0.5) mmol·L^−1^] and CWI_C_ [AIR session: 5.6 (0.4) mmol·L^−1^, N_2_O session: 6.0 (0.3) mmol·L^−1^]. Glucose was reduced (*P* < 0.001), to a similar extent (*P* = 0.17), in all CWIs [AIR session: CWI_A_: 4.3 (0.5) mmol·L^−1^, CWI_B_: 4.1 (0.4) mmol·L^−1^, CWI_C_: 4.1 (0.4) mmol·L^−1^; N_2_O session: CWI_A_: 4.6 (0.8) mmol·L^−1^, CWI_B_: 4.5 (0.5) mmol·L^−1^, CWI_C_: 4.3 (0.5) mmol·L^−1^].

Body mass was reduced by ∼2% (*P* < 0.001) at the end of both sessions [AIR session: −1.4 (0.8) kg, N_2_O session: −1.3 (0.5) kg; *P* = 0.38].

### Thermal Responses

**Figure 2.**
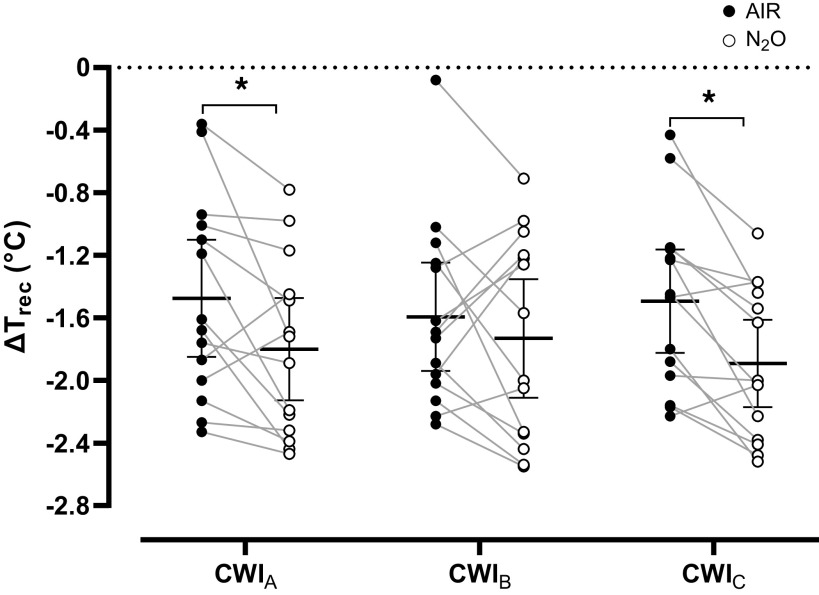
Mean (SD) and individual values of changes in rectal temperature relative to baseline (ΔT_rec_) obtained during the three repeated cold-water immersions (CWIs; 1st CWI: CWI_A_, 2nd CWI: CWI_B_, 3rd CWI: CWI_C_), while subjects (*n* = 14 men) were breathing either room air (AIR) or a normoxic gas mixture containing 30% nitrous oxide (N_2_O). Data were analyzed with a two-way repeated measures ANOVA, followed by Bonferroni post hoc test (*P* < 0.05). *Significant difference between AIR and N_2_O.

Baseline T_rec_ was similar in all CWIs [AIR session: CWI_A_: 37.1 (0.3)°C, CWI_B_: 37.2 (0.3)°C, CWI_C_: 37.1 (0.2)°C; N_2_O session: CWI_A_: 37.1 (0.2)°C, CWI_B_: 37.2 (0.2)°C, CWI_C_: 37.2 (0.3)°C; *P* = 0.65]. N_2_O potentiated the cold-induced reduction in T_rec_ (*P* = 0.01, η_p_^2^ = 0.42), especially during CWI_A_ (*P* = 0.01, *d* = 0.76) and CWI_C_ (*P* = 0.003, *d* = 0.98; Fig. 2). Likewise, N_2_O accelerated the cooling rate in CWI_A_ (AIR: −0.36°C·h^−1^, N_2_O: −0.50°C·h^−1^; *P* = 0.04, *d* = 0.61) and CWI_C_ (AIR: −0.41°C·h^−1^, N_2_O: −0.59°C·h^−1^; *P* = 0.002, *d* = 1.02) but not in CWI_B_ (AIR: −0.45°C·h^−1^, N_2_O: −0.47°C·h^−1^; *P* = 0.78, *d* = 0.07). No intrasession differences in ΔT_rec_ (*P* = 0.20) or cooling rate (*P* = 0.11) were noted.

In both sessions, baseline T_sk_ was slightly higher in CWI_A_ than in CWI_B_ and CWI_C_ [AIR session: CWI_A_: 32.8 (0.6)°C, CWI_B_: 32.0 (0.7)°C, CWI_C_: 32.1 (0.6)°C; N_2_O session: CWI_A_: 32.9 (0.4)°C, CWI_B_: 32.7 (0.5)°C, CWI_C_: 32.0 (0.6)°C; *P* < 0.05]. Baseline T_sk_ was higher by ∼0.7°C in N_2_O than in AIR CWI_B_ (*P* = 0.003). Overall, N_2_O did not alter the cold-induced drop in T_sk_ [AIR session: CWI_A_: 24.3 (0.6)°C, CWI_B_: 24.5 (0.6)°C, CWI_C_: 24.5 (0.8)°C; N_2_O session: CWI_A_: 24.0 (0.7)°C, CWI_B_: 24.3 (0.6)°C, CWI_C_: 24.1 (0.6)°C; *P* = 0.30; η_p_^2^ = 0.08].

### Cardiorespiratory Responses

**Figure 3.**
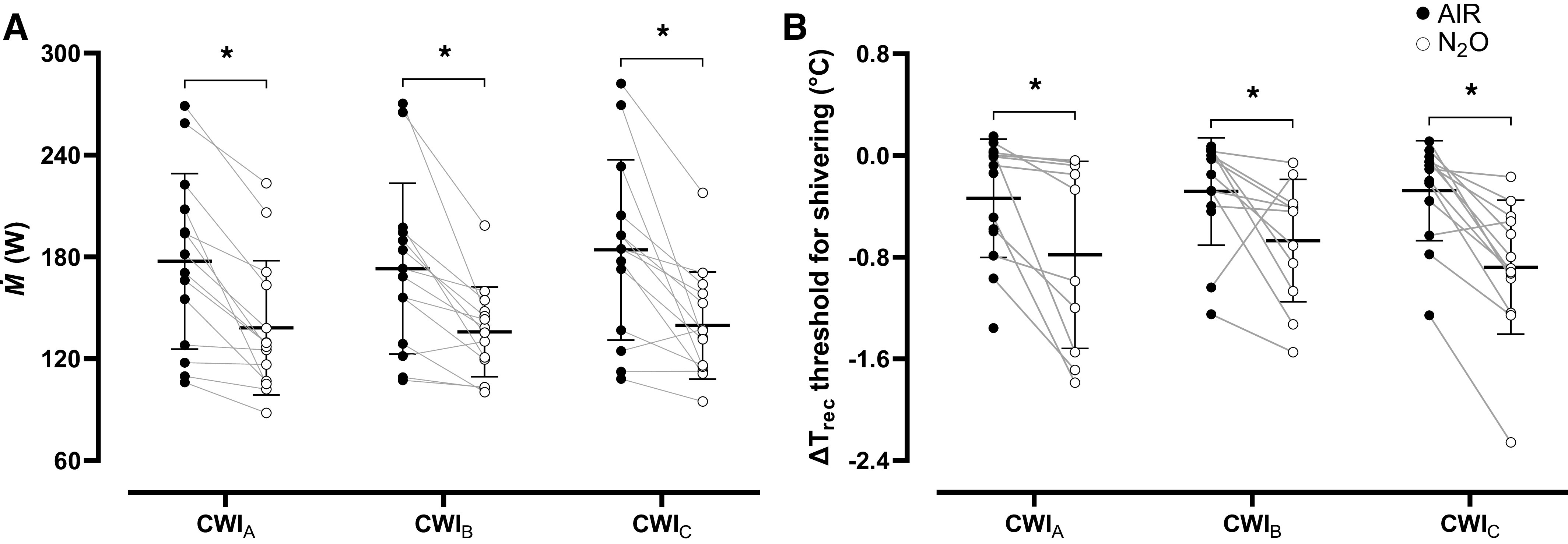
Mean (SD) and individual values of metabolic heat production (*Ṁ*; *A*), and of the shivering thresholds (*B*) obtained during the three repeated cold-water immersions (CWIs; 1st CWI: CWI_A_, 2nd CWI: CWI_B_, 3rd CWI: CWI_C_), while subjects (*n* = 14 men) were breathing either room air (AIR) or a normoxic gas mixture containing 30% nitrous oxide (N_2_O). Data were analyzed with a two-way repeated measures ANOVA, followed by Bonferroni post hoc test (*P* < 0.05). *Significant difference between AIR and N_2_O. ΔT_rec_, relative changes in rectal temperature.

*Ṁ* was similar across the baseline phases [AIR session: CWI_A_: 110 (18) W, CWI_B_: 117 (25) W, CWI_C_: 119 (32) W; N_2_O session: CWI_A_: 121 (21) W, CWI_B_: 108 (14) W, CWI_C_: 115 (17) W; *P* > 0.05]. The cold-induced increase (*P* < 0.001) in *Ṁ* was, in all cases, blunted by N_2_O (*P* < 0.001; η_p_^2^ = 0.63; Fig. 3*A*). N_2_O shifted the threshold for shivering toward a lower ΔT_rec_ (*P* = 0.002; η_p_^2^ = 0.69), especially in CWI_A_ and CWI_C_ (Fig. 3*B*). The number of subjects that exhibited a shivering response in CWI_A_, CWI_B_, and CWI_C_ was, respectively, 14, 13, and 13 in the AIR session and 10, 11, and 13 in the N_2_O session. Subjects also perceived that they shivered less while breathing N_2_O [mean (range) in AIR session: CWI_A_: 2.2 (1–4), CWI_B_: 1.9 (1–4), CWI_C_: 2.1 (1–4); N_2_O session: CWI_A_: 1.5 (1–4), CWI_B_: 1.4 (1–4), CWI_C_: 1.4 (1–4); *P* < 0.001]. N_2_O invariably attenuated the cold-induced elevation in V̇o_2_ (*P* < 0.001; η_p_^2^ = 0.62) and V̇i (*P* = 0.002; η_p_^2^ = 0.54), whereas it did not alter RER (*P* = 0.77; Table 1).

**Table 1.** Cardiorespiratory responses obtained during the three repeated cold-water immersions, while subjects were breathing either room air or a normoxic gas mixture containing 30% nitrous oxide

	CWI_A_		CWI_B_		CWI_C_	
	AIR	N_2_O	AIR	N_2_O	AIR	N_2_O
V̇o_2_, L·min^−1^	0.52 (0.15)	0.41 (0.12)*	0.51 (0.15)	0.40 (0.08)*	0.55 (0.16)‡	0.42 (0.10)*
V̇co_2_, L·min^−1^	0.46 (0.13)	0.34 (0.09)*	0.44 (0.12)	0.33 (0.07)*	0.46 (0.12)	0.34 (0.09)*
V̇i, L·min^−1^	12.0 (4.0)	9.5 (2.2)*	11.1 (3.2)	8.9 (1.6)*	11.7 (3.3)	9.1 (1.5)*
RER	0.85 (0.04)	0.84 (0.17)	0.82 (0.03)†	0.80 (0.09)	0.79 (0.08)†	0.80 (0.09)
EE, kcal	292 (94)	218 (87)*	275 (86)	206 (44)*	295 (99)	213 (71)*
SAP, mmHg	141 (14)	133 (15)*	138 (15)	132 (17)	136 (13)	138 (14)†
DAP, mmHg	89 (9)	83 (9)*	87 (8)	82 (10)	87 (8)	88 (10)

Values are mean (SD) for oxygen uptake (V̇o_2_), carbon dioxide production (V̇co_2_), inspiratory minute ventilation (V̇i), respiratory exchange ratio (RER), energy expenditure (EE), systolic arterial pressure (SAP), and diastolic arterial pressure (DAP). (*n* = 14 men). Data were analyzed with a two-way repeated measures ANOVA, followed by Bonferroni post hoc test (*P* < 0.05). AIR, normal air; CWI_A_, 1st cold-water immersion; CWI_B_, 2nd cold-water immersion; CWI_C_, 3rd cold-water immersion; N_2_O, nitrous oxide. *Significantly different from AIR. †Significantly different from CWI_A_. ‡Significantly different from CWI_B_.

**Figure 4.**
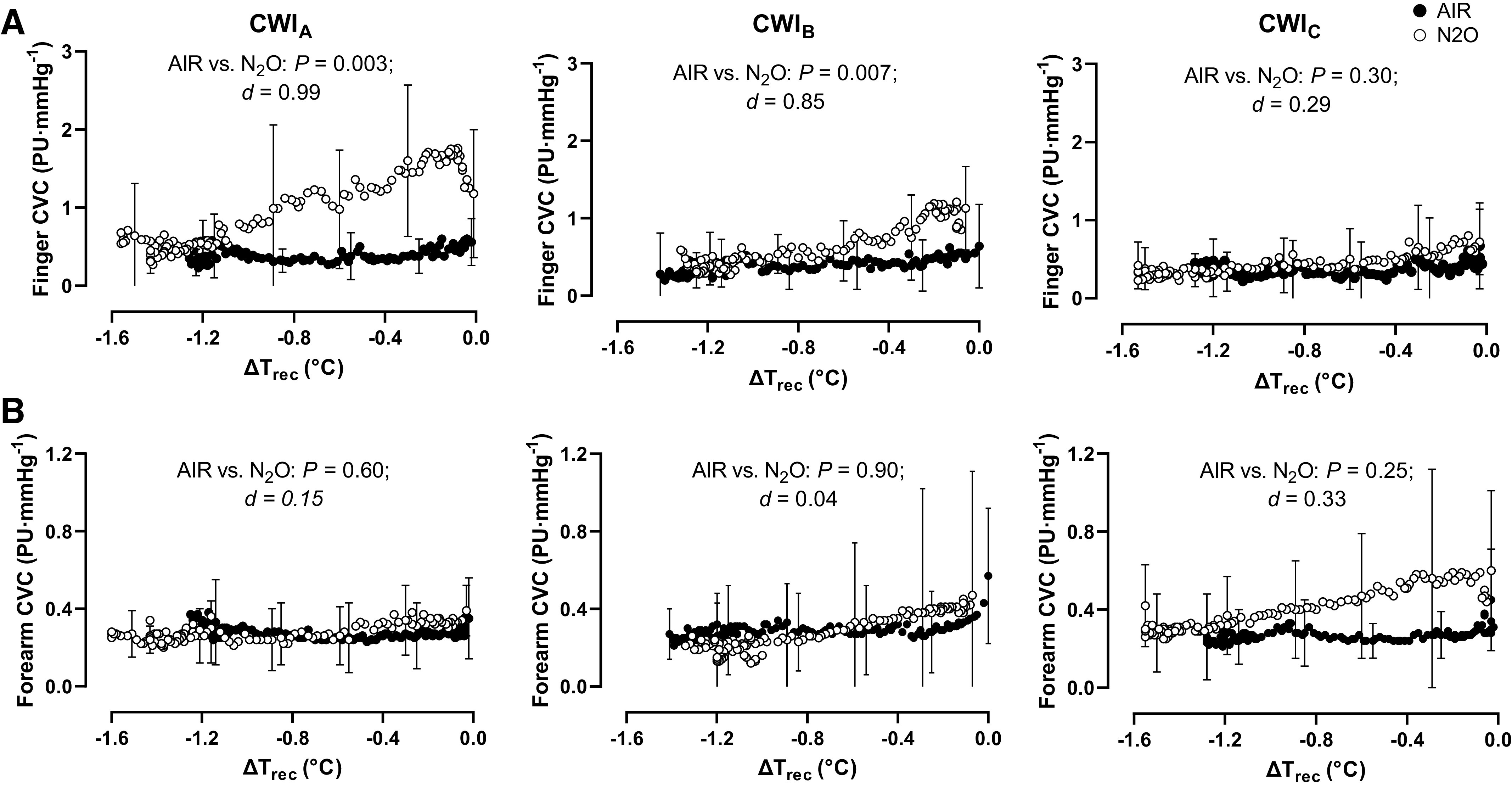
Mean (SD) values of index finger (*A*; *n* = 14 men) and forearm (*B*; *n* = 13 men) cutaneous vascular conductance (CVC) as a function of the relative changes in rectal temperature (ΔT_rec_) obtained during the three repeated cold-water immersions (CWIs; 1st CWI: CWI_A_, 2nd CWI: CWI_B_, 3rd CWI: CWI_C_), while subjects were breathing either room air (AIR) or a normoxic gas mixture containing 30% nitrous oxide (N_2_O). Data were analyzed with a two-way repeated measures ANOVA, followed by Bonferroni post hoc test (*P* < 0.05). Cohen’s *d* was calculated to assess effect sizes, wherein values of ≤0.2, ≤0.5, ≥0.8 were considered small, moderate, and large, respectively.

N_2_O enhanced finger CVC during the baseline phase of CWI_B_ [AIR session: 1.9 (1.4) mmHg·PU^−1^, N_2_O session: 3.2 (0.9) mmHg·PU^−1^; *P* < 0.001] and CWI_C_ [AIR session: 1.9 (1.4) mmHg·PU^−1^, N_2_O session: 2.8 (1.0) mmHg·PU^−1^; *P* = 0.01] but not of CWI_A_ [AIR session: 2.9 (1.1) mmHg·PU^−1^, N_2_O session: 3.3 (0.8) mmHg·PU^−1^; *P* = 0.13). N_2_O attenuated the cold-induced drop in finger CVC during CWI_A_ (*P* = 0.003, *d* = 0.99) and CWI_B_ (*P* = 0.07, *d* = 0.85) but not during CWI_C_ (*P* = 0.30, *d* = 0.29; Fig. 4*A*). Specifically, during the first 30 min of the CWI_A_ and CWI_B_, N_2_O blunted the CVC reduction by ∼63% and ∼25%, respectively (*P* ≤ 0.001). Regardless of the gaseous environment, finger CVC was lower by ∼44% and ∼24% in CWI_C_ than in CWI_A_ (*P* = 0.01) and CWI_B_ (*P* = 0.04), respectively.

No inter- or intrasession differences (*P* > 0.05) were noted as regards the forearm CVC, either during baseline [AIR session: CWI_A_: 0.3 (0.2) mmHg·PU^−1^, CWI_B_: 0.5 (0.5) mmHg·PU^−1^, CWI_C_: 0.4 (0.3) mmHg·PU^−1^; N_2_O session: CWI_A_: 0.7 (1.3) mmHg·PU^−1^, CWI_B_: 0.8 (1.1) mmHg·PU^−1^, CWI_C_: 1.0 (1.1) mmHg·PU^−1^] or CWIs (Fig. 4*B*).

**Figure 5.**
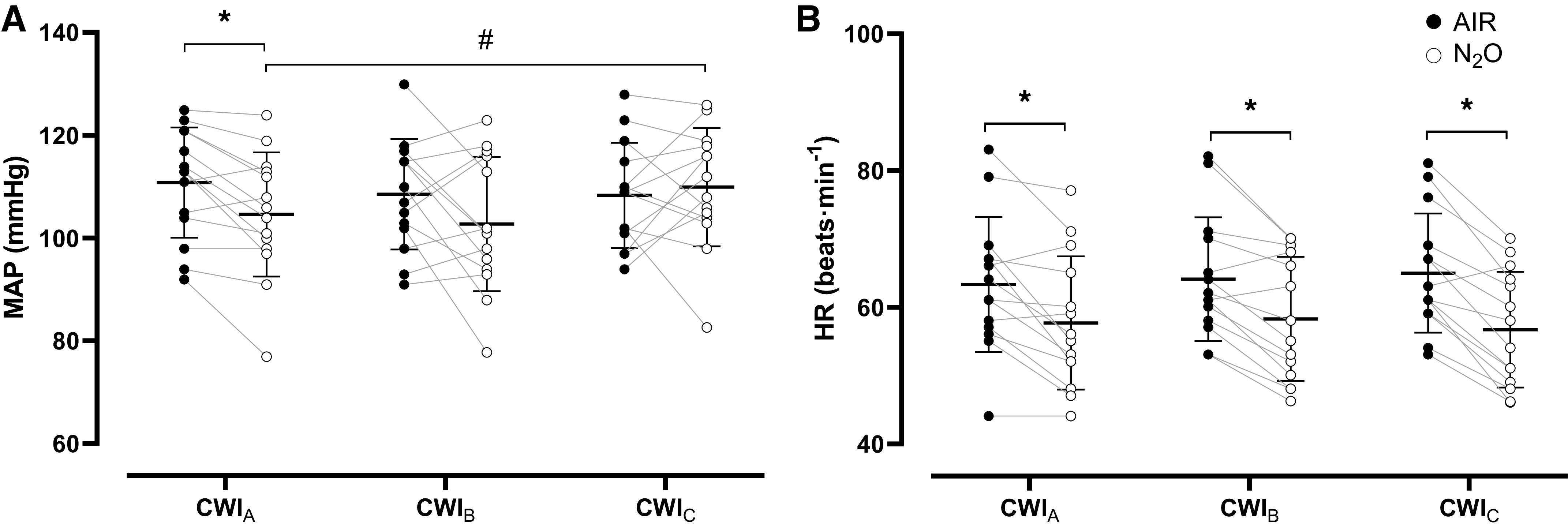
Mean (SD) and individual values of mean arterial pressure (MAP; *A*) and heart rate (HR; *B*) obtained during the three repeated cold-water immersions (CWIs; 1st CWI: CWI_A_, 2nd CWI: CWI_B_, 3rd CWI: CWI_C_), while subjects (*n* = 14 men) were breathing either room air (AIR) or a normoxic gas mixture containing 30% nitrous oxide (N_2_O). Data were analyzed with a two-way repeated measures ANOVA, followed by Bonferroni post hoc test (*P* < 0.05). *Significant difference between AIR and N_2_O. #Significant difference between CWI_A_ and CWI_C_.

During the baseline phases, neither MAP [AIR session: CWI_A_: 99 (11) mmHg, CWI_B_: 99 (11) mmHg, CWI_C_: 103 (9) mmHg; N_2_O session: CWI_A_: 99 (11) mmHg, CWI_B_: 100 (12) mmHg, CWI_C_: 101 (10) mmHg; *P* > 0.05] nor HR [AIR session: CWI_A_: 68 (13) beats·min^−1^, CWI_B_: 76 (14) beats·min^−1^, CWI_C_: 74 (13) beats·min^−1^; N_2_O session: CWI_A_: 70 (11) beats·min^−1^, CWI_B_: 70 (10) beats·min^−1^, CWI_C_: 71 (10) beats·min^−1^; *P* > 0.05] differed. N_2_O blunted the cold-evoked increase in MAP during CWI_A_ (*P* = 0.003, *d* = 0.98; Fig. 5*A*). In the N_2_O session, MAP was lower in CWI_A_ than in CWI_C_ (*P* = 0.04). HR was consistently lower in N_2_O than in AIR CWIs (*P* < 0.001, η_p_^2^ = 0.65; Fig. 5*B*).

### Perceptual Responses

**Figure 6.**
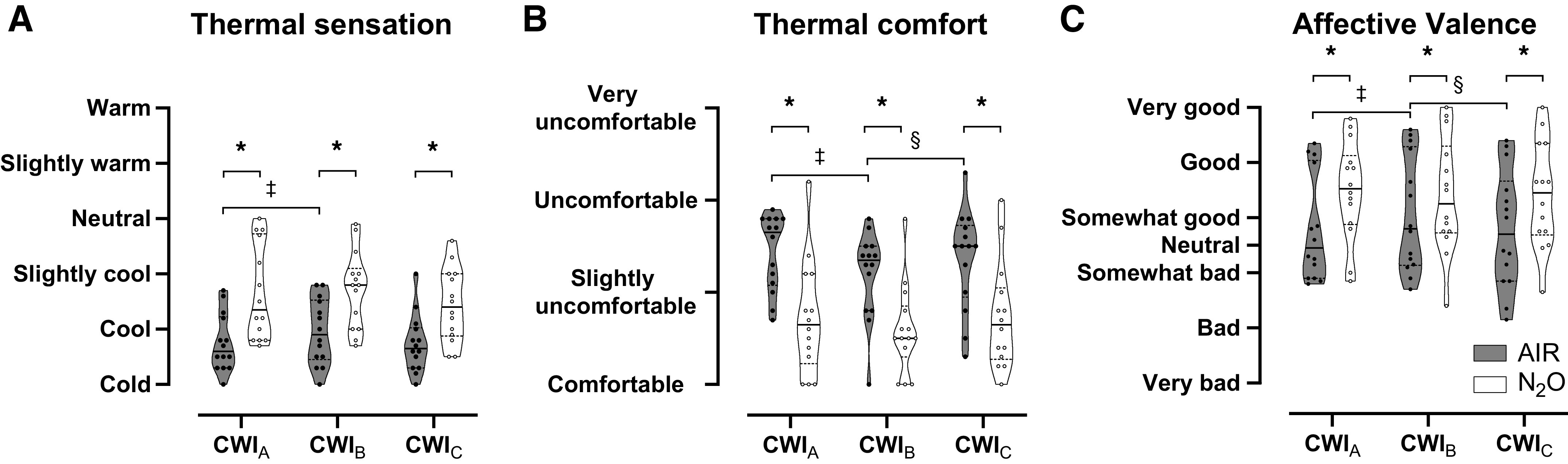
Violin plots of thermal sensation (*A*) and comfort (*B*), and affective valence (*C*) obtained during the three repeated cold-water immersions (CWIs; 1st CWI: CWI_A_, 2nd CWI: CWI_B_, 3rd CWI: CWI_C_), while subjects (*n* = 14 men) were breathing either room air (AIR) or a normoxic gas mixture containing 30% nitrous oxide (N_2_O). The horizontal solid and dotted lines represent median and quartiles, respectively. Data were analyzed with Friedman’s test, followed by the Wilcoxon signed-rank test (*P* < 0.05). *Significant difference between AIR and N_2_O. ‡Significant difference between CWI_A_ and CWI_B_. §Significant difference between CWI_B_ and CWI_C_.

Thermal and affective perceptions did not vary (*P* > 0.05) across the baseline phases [mean (range) for thermal sensation: 5 (4–6); thermal comfort: 1 (1–2); affective valence: 3 (0–5)]. In all CWIs, N_2_O attenuated the sensation of coldness and thermal discomfort (*P* < 0.05; Fig. 6, *A* and *B*). Also, the rates of affective valence were persistently higher in N_2_O than in AIR CWIs (*P* < 0.05; Fig. 6*C*).

## DISCUSSION

It is well documented that short-term administration of a subanesthetic dose of N_2_O impinges on the thermoregulatory system, and potentiates the development of hypothermia during cold stress (3–5). Based on recurrent evidence from animal studies (8–12), the present work sought to evaluate the hypothesis that, in healthy humans, repetitive exposures to N_2_O would gradually diminish the hypothermic response prompted by acute N_2_O. In line with previous findings, a 2-h exposure to 30% N_2_O (i.e., in CWI_A_) blunted thermogenesis and thermoperception, partly attenuated cutaneous vasoconstriction (especially in acral skin), and accelerated body core cooling. However, contrary to our hypothesis, repeated N_2_O exposures within a ∼10-h period failed to alleviate the impairment in the cold-defense thermoeffector activity instigated by acute N_2_O. Present results, therefore, demonstrate that, in humans, N_2_O-induced narcosis jeopardizes the capacity to maintain thermal balance during cold, regardless of the frequency and/or the cumulative duration of the gas exposure.

Our findings complement previous reports (3–5) showing that, in N_2_O conditions, the enhanced rate of body core cooling was predominantly driven by the inability to generate sufficient amounts of endogenous heat. Thus, N_2_O attenuated the cold-induced elevation in $\dot{M}$ by ∼22%–25% and shifted downwards the core temperature threshold for shivering by ∼0.3°C–0.6°C. Notably, the magnitude of metabolic downregulation and shivering inhibition prevailed almost equivalently across the three N_2_O-cold provocations. Such a response is in conflict with the pattern of adaptation described in small homeothermic animals (rats), in which the metabolic responsiveness was progressively restored, and even amplified over prolonged continuous (i.e., ≥3 h; 9) or repeated (i.e., ≥3 daily 3-h sessions; 25) N_2_O exposure.

The present experimental design does not allow us to identify which component(s) of the neural network controlling heat-producing thermoeffectors was (were) affected by N_2_O. Previous studies have shown that N_2_O affects insignificantly cutaneous thermoreception and axonal conduction (26) and probably does not impair the oxidative capacity of skeletal muscle (i.e., the specific effector end-organ; 27). Considering that N_2_O disrupts the functioning of synaptic transmission (28), primarily via the inhibition of the glutamate *N*-methyl-d-aspartate (NMDA) receptors (29–32), it is reasonable to assume that the N_2_O-evoked metabolic downregulation was determined largely by a diminished thermoafferent input (33, 34) and/or a modified central integration of the input (35, 36). Further, we cannot exclude that N_2_O suppressed the descending neural output to the shivering-engaged muscles (37, 38), contributing partly to the response. The sustained $\dot{M}$ attenuation during the N_2_O provocations cannot be ascribed to nonshivering thermogenic processes, given that the metabolism of brown adipose tissue does not appear to be perturbed by acute nor serial N_2_O administration (39, 40). That the reduction in $\dot{M}$ reflected fully a suppressant action of N_2_O on shivering, was also supported by our observation that overt tremor was initiated or intensified promptly after the subjects were reinstated to the room-air breathing conditions during the rewarming phase; this was also corroborated by most of the subjects informally reporting enhanced shivering activity, as well as augmented sensations of coldness and discomfort during this period.

Along with the reduction in heat production, N_2_O appeared to also modulate, to some degree, heat conservation. In line with a previous observation (5), N_2_O enhanced cutaneous circulation already during the baseline phase, while subjects were euthermic. Interestingly, and contrary to Passias et al. (5), wherein the N_2_O-dependent alterations in skin perfusion were ostensibly abolished immediately upon immersion in 15°C water, the response was prevalent during the initial portion (i.e., until ∼30 min) of the 20°C-CWIs, attenuating the magnitude of cold-induced vasoconstriction. Yet, after this period, and when subjects were rendered mildly hypothermic (greater than ∼0.05°C drop in T_rec_; see Fig. 4), the N_2_O-evoked influence on cutaneous vasomotion was overridden apparently by the strong vasoconstrictive drive instigated by the deep-body cooling. It has previously been suggested that N_2_O might increase peripheral blood flow via upregulation of nitric oxide—i.e., a potent vasodilator (9). To the best of our knowledge, no direct evidence exists, however, that, in humans, N_2_O augments nitric-oxide production and bioavailability, systemically or locally. Furthermore, considering that, in the current study, N_2_O enhanced flow exclusively in the acral skin vasculature (the palmar side of the finger), which is predominantly regulated by the noradrenergic vasoconstrictor system (41, 42), it seems unlikely that the nitric-oxide vasodilator system may have played a role. Rather, the diminished constrictor response in finger arterioles was probably attributable to the N_2_O-mediated attenuation of reflex sympathetic activation, as also indicated by the blunted cold-induced elevations in HR and V̇i, and, at least during CWI_A_, in MAP. It is noteworthy, however, that the inhibitory potency of N_2_O on acral vasoactivity faded over the successive immersions, presumably due to nonthermoregulatory influences, such as the modulations in central blood volume evoked by the repeated immersions in and egressions from the (cold and warm) water (43–46), as well as the changes in subjects’ emotional state conceivably associated with the development of some level of mental fatigue (47, 48). Regardless of the mechanism, the gradual attenuation of the N_2_O-induced impairment on the heat-conservation processes may also explain the lack of intersession difference in ΔT_rec_ during the CWI_B_ (at least statistically, because numerically it was still ∼0.2°C lower in N_2_O than in AIR).

N_2_O alleviated the sensation of coldness and thermal discomfort; a finding that conforms to those from previous investigations (3, 5, 49). In support of our hypothesis, the N_2_O-evoked alliesthetic responses remained unaltered across the three repeated CWIs. The persistent attenuation of the discriminative and hedonic perceptions emerged despite the more prominent hypothermic response obtained in the N_2_O CWIs, and independently of the thermal inputs from the skin (i.e., T_sk_ did not vary between the two gaseous environments). Presumably, the thermoperceptual desensitization can be ascribed to direct actions of N_2_O on sites of the central nervous system that are responsible for the integration and processing of thermosensory information (50–52), thereby modulating the thermoperceptual output. That the cognitive and sensory attributes may also have been influenced partly by the subjects’ more positive generalized affective state (53), as well as by their self-awareness regarding the reduced shivering tremor (54) during the N_2_O trials, can neither be excluded. Further, the perceptual ratings might arguably have been confounded, at least to a degree, by a placebo effect, given that almost all subjects were able to identify the gas inspired. Finally, and regardless of the origin of the response, it remains unsettled whether, in conditions of behavioral freedom, the N_2_O-evoked thermoperceptual impairments would alter the desire for and the efficacy of consciously recruiting thermos-behavioral counteractions. For instance, although the cold-defense behavior is indeed compromised in animals (e.g., they seek for cooler ambiance; 10, 55, 56), Yogev and Mekjavic (57), by utilizing an operant conditioning protocol in humans, failed to detect any interference of the N_2_O-induced alleviation of thermal discomfort on the capacity to thermoregulate behaviorally.

Collectively, current results demonstrate that, in response to a prolonged iterative cold stimulus, N_2_O-induced narcosis impairs in a persistent manner the capacity to activate heat-generating thermoeffectors and to consciously assess the intensity and (un)pleasantness of the thermal stimulus. In view of our findings, as well as of those of Cheung and Mekjavic (3) that evaluated the impact of varying doses (ranging from 10% to 25%) of N_2_O, it might hence be deduced that the influence of subanesthetic amounts of N_2_O on human autonomic thermoregulation is probably relatively independent of the dosage (i.e., partial pressure and frequency) of the gas. By contrast, the thermoperceptual responses, although they were not affected by the cumulative period of gas exposure in this study, seem to be determined by the intensity of the gas (3) and thermal (49) stresses imposed. Still, whether N_2_O administration of longer duration (e.g., sustained exposure of >2 h or repeated exposures over several days) and/or of higher severity (i.e., >30%) would induce a different pattern of thermoadaptation, remains unclear. It should however be taken into account that the employment of prolonged continuous or severer exposure to N_2_O may enhance the risk of neurotoxicity (18, 19).

### Methodological Considerations

The previous investigations in rats have described a high interindividual variability with regards to the pattern of adaptation evoked by repeated N_2_O administration, suggesting that the mode of response is determined largely by the animal’s sensitivity to the initial N_2_O exposure (9, 58). That is, heat production was gradually upregulated over exposure time, mainly in the rats that were insensitive (i.e., exhibited a smaller metabolic downregulation) to acute N_2_O. Of interest is that, in the present study, the cold-induced elevation in $\dot{M}$ was higher during the N_2_O than the AIR CWI_B_ and CWI_C_ in only one individual, who did present the smaller $\dot{M}$ attenuation during the N_2_O CWI_A_ (see Fig. 3*A*). Considering that the cohort of subjects tested herein described similar biological (e.g., age, sex) and morphological characteristics, and previous experience with N_2_O, we are unable to identify the nature of interindividual variation on the magnitude and direction of thermoeffector responses to acute and repeated N_2_O exposure. Nevertheless, current results point to the fact that, as regards thermoregulation, extrapolation of results from animal studies to humans must be performed with caution.

We used immersions in 20°C water (i.e., a high heat-loss environment) to provoke, safely and relatively shortly, adequate degrees of deep-body cooling, thereby triggering moderate levels of shivering activity. Previous studies have shown that three serial cold-water immersions within a single day led to a progressive hypothermic response, described by metabolic and thermoperceptual habituation (13, 14). In the present study, however, no such thermoadaptation to repetitive cold stimuli (i.e., in the AIR CWIs) was confirmed, aside from a transient mitigation of the temperature-related sensations during CWI_B_ (see Fig. 6). The reason for the inconsistent results in the present and the previous studies is not clear.

In both sessions, the baseline values of blood glucose were slightly lower in the morning (i.e., CWI_A_) than in the afternoon CWIs, probably reflecting its diurnal variation pattern (see Ref. 59). It is, however, highly unlikely that these small differences in glucose may have influenced the thermoregulatory responses to cold, given that subjects remained euglycemic throughout (i.e., glucose was 4.0–6.0 mmol·L^−1^; cf. Refs. 60, 61). Also, to eliminate the confounding influence of circadian rhythmicity, the serial immersions were performed at the same time of the day in the two experimental sessions. The similar reduction in subjects’ body weight at the end of each session indicates that the magnitude of body fluid loss did not differ between the AIR and N_2_O sessions; yet the manner in which N_2_O and hypovolemia might interact presumably affecting vasomotor responses to cold remains unclear. Further, the study would have benefited from the use of a more dynamically responsive index of deep-body temperature (e.g., esophageal temperature; cf. Ref. 62), the monitoring of temperature at additional skin sites, as well as the measurement of hormone and metabolite levels. Finally, future work should determine whether sex differences exist in thermoeffector responses to inert gas narcosis.

### Conclusions

Present results demonstrate that, regardless of the duration of gaseous exposure, human thermoregulatory function is jeopardized by N_2_O-induced narcosis. Thus, acute, as well as repeated exposure to 30% N_2_O blunts consistently shivering-thermogenesis and thermoperception, partly attenuates cold-induced vasoconstriction, and potentiates hypothermia. From a practical perspective, current findings further substantiate that inert gas narcosis constitutes a predisposing factor to accidental hypothermia during short-term, as well as prolonged and/or repeated dives in cold water. Further investigation is needed to elucidate the mechanisms underlying the thermoadaptive modifications elicited by inert gas narcosis.

## DATA AVAILABILITY

Data will be made available upon reasonable request.

## GRANTS

The study was funded by the Swedish Armed Forces (Grant No. AF. 9220919); M.E.K. was supported by a salary grant from the KTH Royal Institute of Technology (Grant No. C-2020-0748).

## DISCLOSURES

No conflicts of interest, financial or otherwise, are declared by the authors.

## AUTHOR CONTRIBUTIONS

M.I.M., A.E., M.G., O.E., and M.E.K. conceived and designed research; M.I.M., A.E., O.E., and M.E.K. performed experiments; M.I.M. analyzed data; M.I.M. and M.E.K. interpreted results of experiments; M.I.M. prepared figures; M.I.M. drafted manuscript; M.I.M., A.E., M.G., O.E., and M.E.K. edited and revised manuscript; M.I.M., A.E., M.G., O.E., and M.E.K. approved final version of manuscript. 
